# Supplementary material for: Molecular traces of Drosophila hemocytes reveal transcriptomic conservation with vertebrate myeloid cells
Source: PLoS Genet. 2023 Dec 19;19(12):e1011077. doi: 10.1371/journal.pgen.1011077 (PMC10763942; doi:10.1371/journal.pgen.1011077)
Supplement: Attachment — Submitted filename: Point-by-point response.pdf [file pgen.1011077.s018.pdf]

Reviewer #1:

This paper presents a novel and systematic comparison of Drosophila immune cells with vertebrate immune cells using single-cell transcriptomics. Drosophila immune cells are often compared with their vertebrate counterparts, and such a comprehensive comparison is therefore much needed. Some of the claims (Drosophila hemocytes are counterparts of primarily vertebrate innate immune cells, PH1 are counterparts of progenitors, and plasmocytes to macrophages) are well supported, but the comparison of Drosophila lamellocytes to neutrophils needs additional information (gene list with cluster information - see below) and discussion. The paper is otherwise written clearly with adequate presentation in figures, the authors have used appropriate tools (I am not familiar with GSVA and MetaNeighbor analyses to assess their use) that are available online, and the original data are deposited in appropriate databases.

The authors have produced a valuable single-cell atlas of larval hemocytes of embryonic and lymph gland origin from different time points during the 3rd larval instar and also during parasitoid wasp infestation. This comprehensive atlas thus complements previous single-cell RNAseq projects of Drosophila hemocytes. All data are available in the online Fly scRNA-seq database, which is certainly a very valuable tool for researchers interested in Drosophila immunity and hematopoiesis, but not only for them.

RE) We thank the reviewer for positive feedback and constructive suggestions. Our manuscript has been modified accordingly, incorporating additional scRNA-seq datasets. Specifically, we analyzed the data from five public *Drosophila* scRNA-seq studies and provided an integrated dataset with clustering in our revised manuscript. In addition to the cell types and states provided by the original papers, we clustered 125,402 cells into 13 transcriptomic states representing six major cell types. Now, the integrated single-cell atlas of Drosophila hemocytes is visualized in our web-based database (http://big.hanyang.ac.kr/flyscrna). Please find the details below.

Major points:

1. The specific genes that are shared by Drosophila and either zebrafish, mouse or human (Fig. 2b) are not listed. Only selected genes that encode CD molecules are listed in Table S1. It would be useful to create a table with all these genes and have

information on their expression in each cluster of the two species being compared or to create a searchable database with this information. For example, lines 318-321: We found that marker genes of Drosophila PH 1 cells, PMs (120 h AEL), and LMs were highly expressed in zebrafish HSCs, macrophages, and neutrophils, respectively, as we observed in the MetaNeighbor analysis (Fig. 4a and Supplementary Fig. 7a)." What genes are mentioned here? Lines 342-345: "Likewise, LMs were related to neutrophils in both zebrafish and mouse, and PMs and adipohemocytes from 120 h AEL larvae showed conservation with vertebrate macrophages or monocytes, illustrating features shared by Drosophila hemocytes and innate immune cells in more complex organisms." What are the specific features or genes common to these cell types?

RE) Thank you for the valuable comment. As the reviewer suggested, we have now included tables for *Drosophila* orthologs, **Supplementary Tables S3** and **S4**, which contain lists of orthologs between *Drosophila* and zebrafish, mice, and humans, as well as 4267 common genes between all four species. The orthologous gene tables are also available through the Fly scRNA database (http://big.hanyang.ac.kr/flyscrna/). It is important to note that it is not feasible to list a specific set of genes from MetaNeighbor analyses as they depend on the transcriptomic similarities between two populations of cells, not just on the expression of a few marker genes. However, enrichment analysis (GSVA) uses predefined marker genes for each cell type. The full list of cell type/state markers is summarized in **Supplementary Table S1,** and the specific genes used in the enrichment analysis can be found in the Fly scRNA database.

2. While PH1 as counterparts of vertebrate progenitors and PM as monocytes/macrophages are more convincing and consistent with previous functional observations (PM are phagocytes, for example), LM are presented throughout the paper as counterparts of neutrophils, but this is not very convincing - they share features with monocytes/macrophages as well (Fig. 5 compared to human, Fig. S9B). As it is now stated in the paper, this could be cited in the future as "lamellocytes are counterparts of neutrophils", which would be very simplified and the authors should ensure that this is not the case. The publication contains no in-depth discussion comparing lamellocytes and neutrophils, their functioning and roles. Lamellocytes may be very specialized cells specific to only certain species of Drosophila. Again, for future studies and interpretation, it would be helpful to have a tool to look at which genes are

actually common to LM and different vertebrate cell types. The authors should look into this comparison in more detail and be very careful in formulating their conclusions.

RE) We agree with the reviewer's concerns. Although we found similarities between lamellocytes and neutrophils in vertebrates, the evidence (for example, marker genes of cell types with certain functions) only weakly associated the natural functions of lamellocytes with those of neutrophils. In the revised manuscript, we removed the statements related to these cell types and discussed possible similarities in the discussions only.

Reviewer #2:

Using their own and published single-cell transcriptomic data, the authors have made an ambitious attempt to trace the relationships between blood cell types in flies and vertebrates. Much of the data are also made easily accessible in a flyscrna database. This is very helpful. The results are not entirely clear-cut, but they should still be of interest for a broad audience. However, there are some problems with the interpretations that the authors have to address before publication.

RE) We thank the reviewer for providing insightful comments and raising valuable concerns. In response, we have conducted additional analyses to offer a broader perspective and thoroughly revised the manuscript. Please find detailed revisions below.

As a starting point, the authors made an integrated clustering re-analysis of their previously published single-cell data from circulating and lymph gland cells (Tattikota et al. 2020 and Cho et al. 2020). Worryingly, the resulting clusters and subclusters are have poor match with similar studies published elsewhere. In total, at least six such studies have been published, four with circulating hemocytes (Cattenoz et al. 2020, Tattikota et al. 2020, Fu et al. 2020 and Leitão et al. 2020) and two with lymph glands (Cho et al. 2020 and Girard et al. 2021). These should all be properly referred to, and the discrepancies must be discussed. The analysis described in this manuscript corresponds well with one of their own studies (Cho et al. 2020), but not with the other one (Tattikota et al. 2020). The other four studies are not even mentioned in this manuscript.

RE) The reviewer has raised a valid concern regarding the matching of clusters from six different studies, including our own. It is important to acknowledge that all these studies used different criteria and parameters [1-6] for their clustering analyses. For example, Cattenoz et al. used *Seurat* to perform a clustering analysis with 20 principal components and a 0.55 resolution, followed by subclustering analyses and manual curation for each cluster [1]. On the other hand, our previous analysis used different parameters (52 principal components, 0.8 resolution) for initial clustering, followed by the aggregation of clusters into broad cell types. Then, each cell type was iteratively clustered, and similar subclusters were aggregated to define heterogeneous cell states (see “optimal subclustering analysis” in the Cho et al.’s Methods section) [2].

To determine the differences between the clusters in each study, we obtained raw scRNA-seq data and cell annotations from public repositories whenever available (**Reviewer's Table 1**). We also received the raw data of Fu et al. from the authors and the cell annotations of Girard et al. from the first author via personal communication. All raw datasets were analyzed using the same genome version (BDGP 6.22, accession code: GCA_000001215.4) to ensure a fair comparison, except for two InDrops samples from Tattikota et al., which had technical issues in the analytic pipeline. For these samples, we downloaded processed count data and updated the gene annotation to be compatible with BDGP6.22 by matching gene IDs.

Dataset	Strain	Origin	Condition	Platform	Accession code
**Cattenoz et al.**	OregonR	Circulation	WT/Infect	10X 3'-seq	E-MATB-8698
**Tattikota et al.**	w1118	Circulation	WT/wound	inDrops/10X 3'-seq	GSE146596
**Fu et al.**	w1118	Circulation	WT	10X 3'-seq	Personal communication
**Leitao et al.**	outbreed	Circulation	WT/Infect	10X 3'-seq	GSE148826
**Girard et al.**	w1118	Lymph gland	WT	10X 3'-seq	GSE168823

Reviewer's Table 1. Public scRNA-seq datasets included in the revised manuscript

All datasets were aligned to a *Drosophila* reference genome and quantified using CellRanger with the reference genome and matching gene annotation. *Seurat* v4 was used to analyze the resulting UMI count matrices.

To filter low-quality cells, library-specific thresholds for gene counts and mitochondrial gene proportions were used.

For Cattenoz et al., only cells with ≥ 500 genes and < 20% mitochondrial genes were included.

For Fu et al., only cells with ≥ 500 genes and < 10% mitochondrial genes were included.

For Girard et al., only cells with ≥ 1500 genes and < 5% mitochondrial genes were included.

For Leitão et al., only cells with ≥ 200 genes and < 40% mitochondrial genes (C1_Uninf, C3_Inf) or 30% mitochondrial genes (others) were included.

For Tattikota et al., cells with ≥ 250 (replicate 1) or 500 (replicate 2) genes and < 25% mitochondrial genes were included for 10X data. For the InDrops data of Tattikota et al., cells with ≥ 500 (replicate 3) or 100 (replicate 4) genes and < 20% mitochondrial genes were included.

Finally, for each sequencing library, cells with UMIs higher than the *mean + 2 standard deviations* were removed (**Reviewer's Fig. 1**).

Reviewer's Figure 1

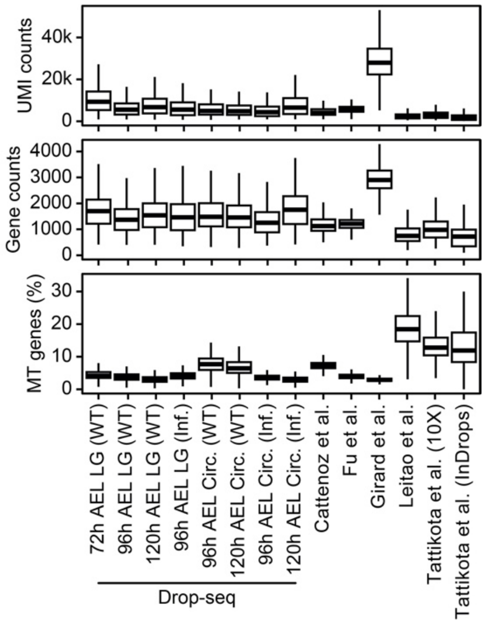

Reviewer's Figure 1. UMI and gene counts and mitochondrial gene proportions (%) in processed public scRNA-seq datasets

The cell annotations of Cattenoz et al., Girard et al., Leitao et al., and Tattikota et al. were assigned by matching barcode sequences, while cells that were additionally included in this study were inferred using label transfer analysis. The scRNA-seq data of Fu et al. was clustered at a resolution of 0.3 and annotated using marker genes reported in the original study (**Reviewer's Fig. 2a**). For each dataset, label transfer analysis was performed to infer the cell types/states annotated in our study (**Reviewer's Fig. 2b**). We found that crystal cells and lamellocytes annotations agreed well between different studies; however, the cell states of plasmatocytes showed high heterogeneity (**Reviewer's Fig. 3a** and **b**). Disagreement in the transcriptomic states of plasmatocytes was also apparent when comparing these studies (**Reviewer's Fig. 4**). Furthermore, the PL-ImpL2 subcluster of Cattenoz et al., which was determined to be expressing *CG15550*, *tau*, *CG10038*, *ImpL2*, and *kn* in the original study, was best matched to PSC cells (**Reviewer's Figures 2b** and **3**). The thanacyte of Fu et al., which expressed *Ance* and *Tep4*, was broadly matched to PH clusters (**Reviewer's Figures 2b** and **3**).

In the revised manuscript, we have included combined cell annotations from other five previous studies to better appreciate the heterogeneous cell types and states. These annotations are presented in **Figure 2** and **Supplementary Figures 3** and **4.** We have also updated this information in the Fly scRNA database (http://big.hanyang.ac.kr/flyscrna/).

Reviewer's Figure 2

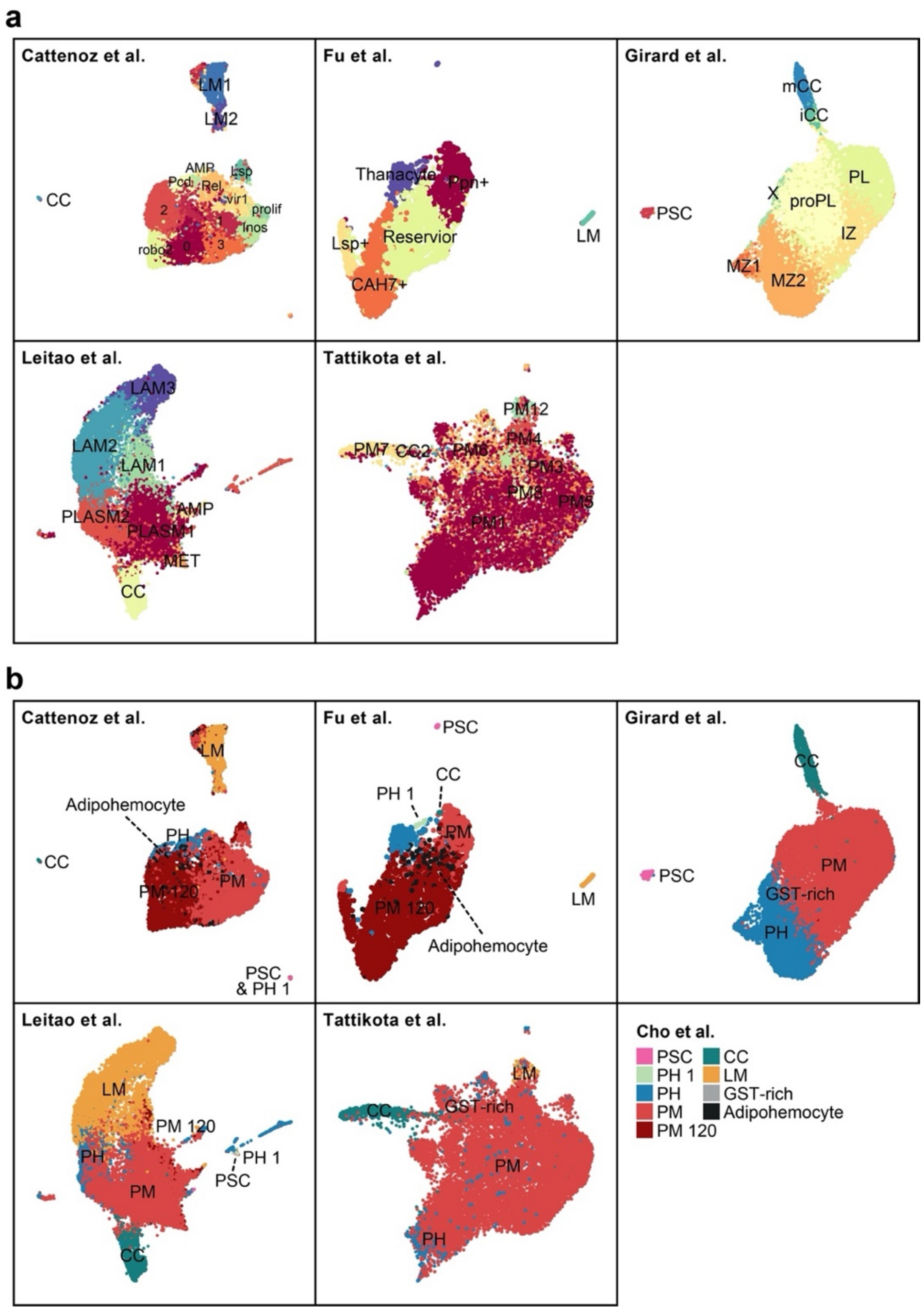

Reviewer's Figure 2. Cell annotations of public scRNA-seq datasets *Annotation using the (**a**) cell types and subtypes reported in the original paper and (**b**) cell types and subtypes predicted by label transfer with the cell type annotations from Cho et al.*

Reviewer's Figure 3

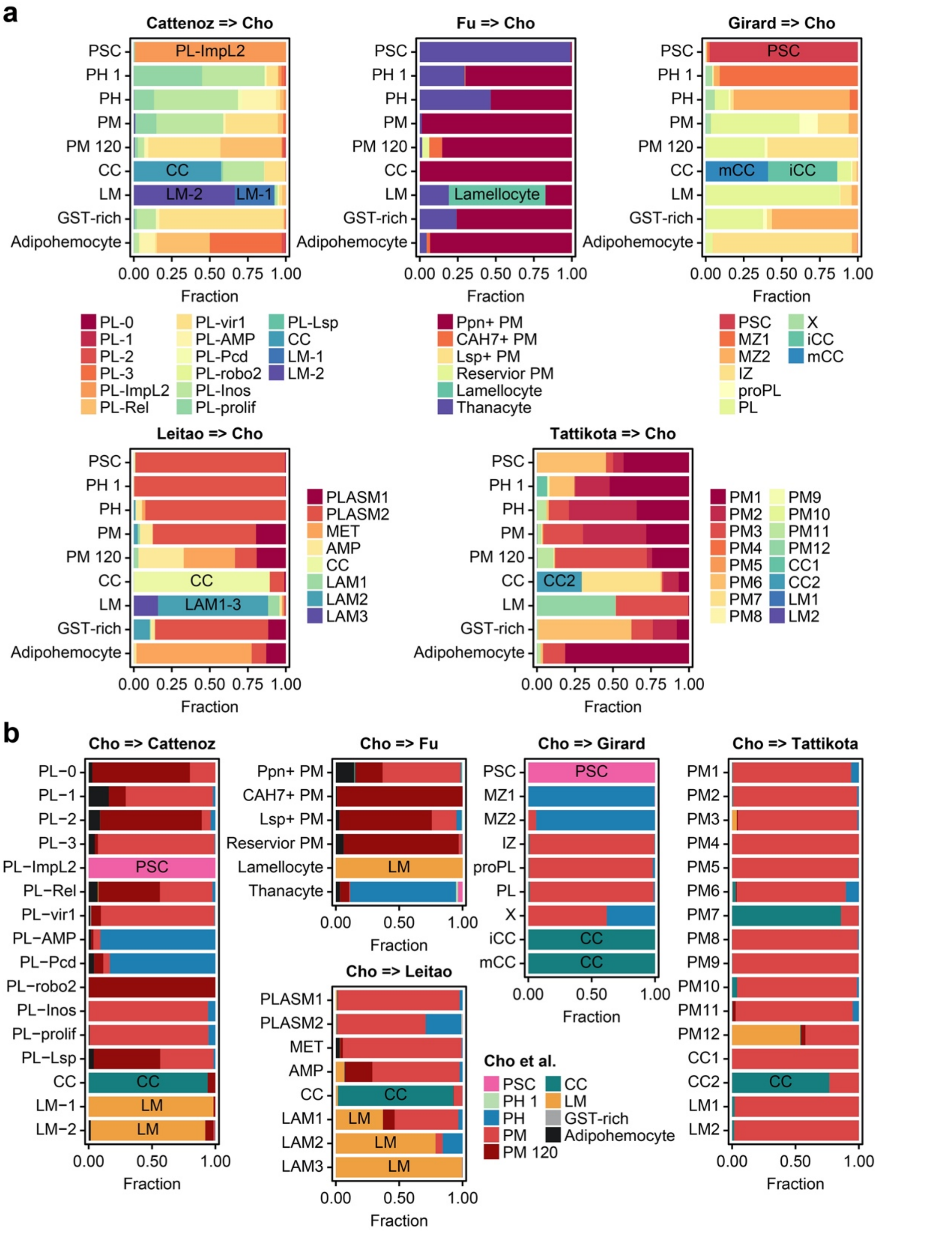

***Reviewer's Figure 3. Predictions of annotations using a label transfer analysis** (**a**) Predictions of cell types/subtypes in Cho et al. using annotations from each of five public scRNA-seq datasets. (**b**) Prediction of cell types/subtypes in each of the five public scRNA-seq datasets by label transfer analysis using the cell type annotations from Cho et al.*

Reviewer's Figure 4

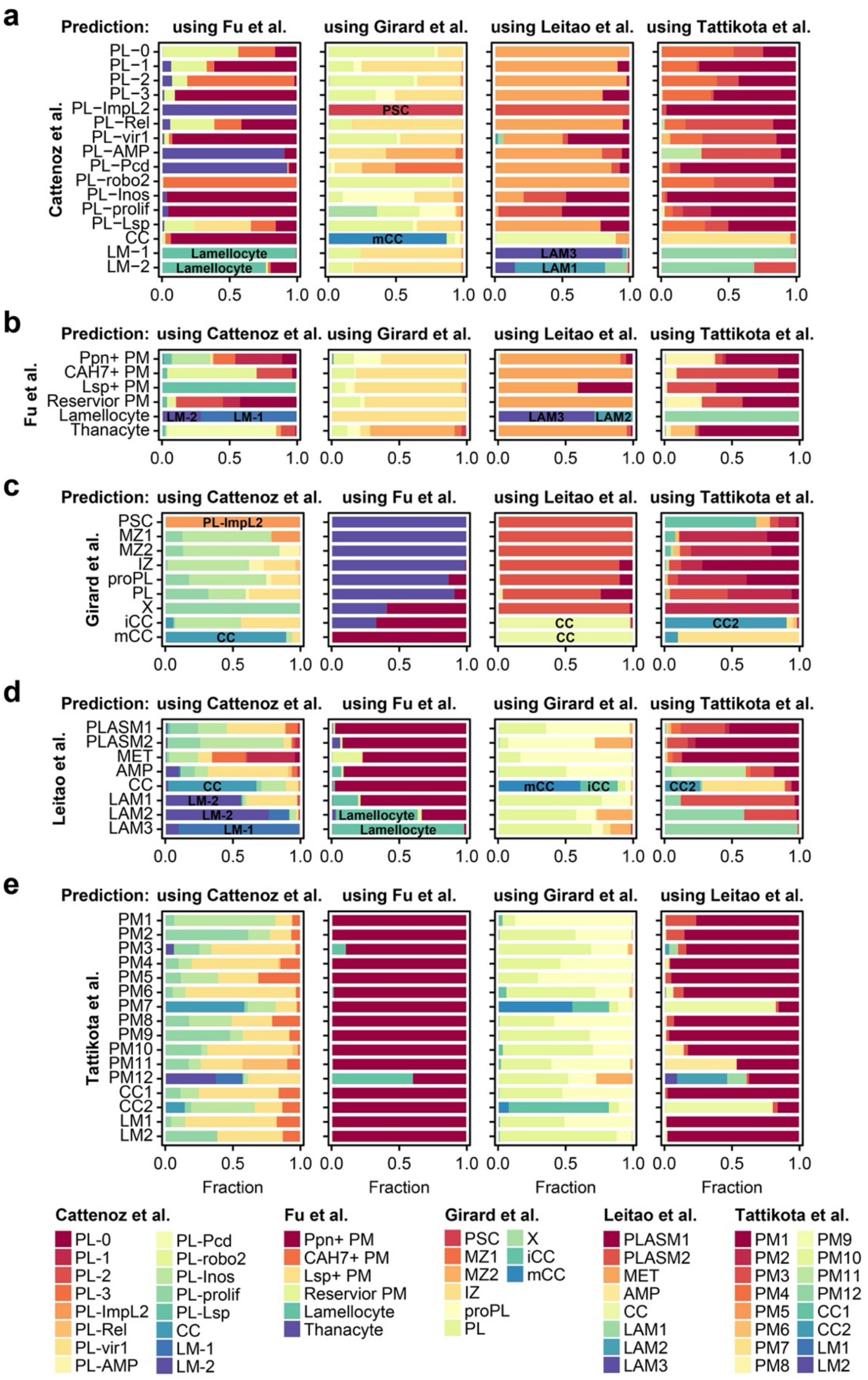

Reviewer's Figure 4. Predictions of cell annotations using label transfer analysis
*Predictions of cell types/subtypes of Cattenoz et al. (**a**), Fu et al. (**b**), Girard et al. (**c**), Leitão et al. (**d**), and Tattikota et al. (**e**) using the annotations of the other four public scRNA-seq datasets.*

For this discussion, it may be constructive to distinguish between cell types and cell states. Cell types are more or less stably differentiated lines of cells. Lamellocytes and crystal cells are such classically defined hemocyte types, and they are nicely supported by all six transcriptomic studies. However, the remaining perhaps 50-95% of the cells are split into various clusters and subclusters, most of them corresponding to the plasmatocyte cell type, but perhaps transiently involved in particular activities, or states, such as mitosis or antimicrobial and stress responses. Specifically, the GST cluster may correspond to cells in a state of stress, and its markers overlap partially with clusters described in Tattikota et al. 2020, and perhaps to a limited extent in other studies as well.

RE) Crystal cells (CCs) and lamellocytes (LMs) were found to be consistent across six different studies, however, plasmatocyte (PM) clusters were defined at different levels in each study (**Reviewer's Figs. 3** and **4**). To better understand the heterogeneous cell types and states in lymph gland and circulating hemocytes, all cells from six scRNA-seq datasets were integrated and re-clustered (**Reviewer's Fig. 5a**, n = 128,542). A total of 17 clusters were identified, and based on marker gene expression and annotations from the previous studies, six major cell types (posterior signaling center [PSC], prohemocyte [PH] 1, PH, PM, LM, and CC) were identified. The PM cell type displayed the highest diversity of transcriptomic states (**Reviewer's Fig. 5a**). Four small clusters were removed in the subsequent analyses, including two clusters from Tattikota et al's InDrops dataset ("Hsp (InDrops)" and "Unknown (InDrops)" in **Reviewer's Fig. 5**), a cluster enriched with muscle-specific marker genes, such as *Mlc1* or *Mlc2* ("Muscle" in **Reviewer's Fig. 5**), and a cluster mostly originating from Leitão et al. that was enriched with male-specific genes, such as *Mst84Da* or *S-Lap7* ("S-Lap (Leitão)" in **Reviewer's Fig. 5**) (**Reviewer's Fig. 5b**). Of the remaining 13 clusters, seven clusters were associated with heterogeneous transcriptomic states of plasmatocytes ranging from proliferation to Lsp-enriched states (**Reviewer's Fig. 5a** and **c**). All cells that were predicted to be GST-rich in the label transfer analysis were included in the PM-Gst cluster, which was characterized by the expression of glutathione S transferases and distinguished from other plasmatocyte states (**Reviewer's Fig. 5c** and **d**). Prohemocytes (PH) were identified by the expression of *Tep4* and *Ance* and were the second most frequently occurring cell type in lymph gland samples, along with PSC cells (**Reviewer's Fig. 5c** and **d**). Most PH cells in circulating hemocytes were identified in wasp-infected larvae (72.24%, 4600 out of 6368 cells),

likely originating from dissociated lymph glands. In general, two PM-late clusters, PM-Lsp, lamellocytes, and crystal cells were mainly found in circulating hemocytes, and both lamellocyte clusters were primarily identified under wasp infection (**Reviewer's Fig. 5d**). We have updated our revised manuscript with these new clustering results and added to the discussion.

Reviewer's Figure 5

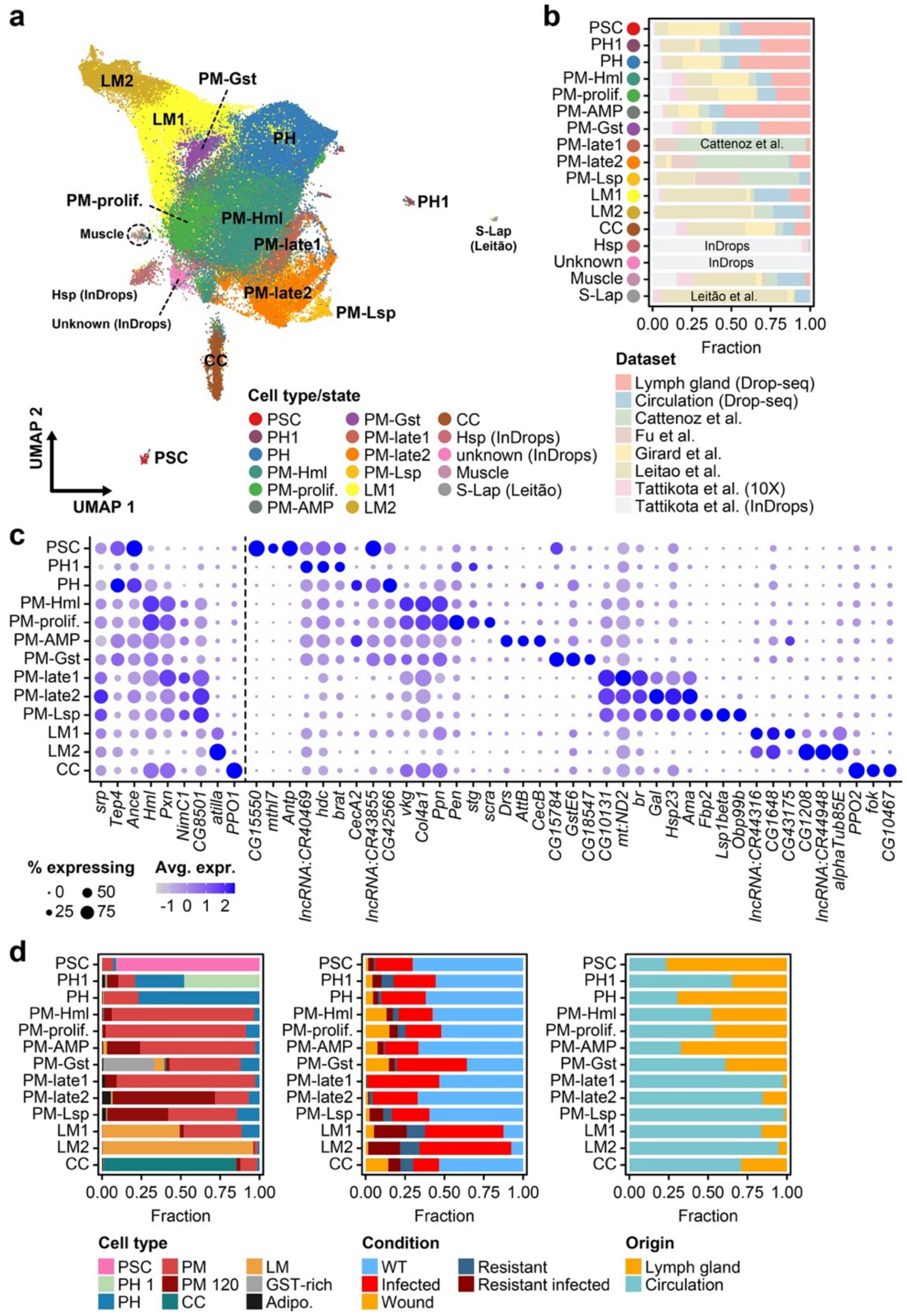

Reviewer's Figure 5. Integrative clustering analysis of six scRNA-seq studies *(****a****) A UMAP plot showing 17 clusters (13 hemocyte and 4 non-hemocyte clusters). (****b****) The*

*proportion that each scRNA-seq dataset contributes to each cluster. (**c**) Expression of canonical cell type markers (left) and the top three marker genes for each hemocyte cluster. (**d**) The proportions of broad cell types for each cell type/state defined in the integrative analysis (left) and categorized by sampling condition (middle) and tissue origin of cells (right).*

The PH (prohemocyte) cluster is a special case. It includes a substantial fraction of all non-lamellocyte and non-crystal cells. Surprisingly, four of the "top 5 cell type markers" for the PH cluster (Fig. 1 d) are antimicrobial peptides, otherwise characterizing minor "AMP" subclusters in the other studies. The fifth marker, CG13160, was only detected in the lymph gland, according to the data in the flyscrna database. By exclusion, the majority of cells in the PH cluster must classically be defined as plasmatocytes, since most or all of the circulating non-lamellocyte and non-crystal cells are known to express classical markers of differentiated plasmatocytes (NimC1, Hml...). If true prohemocytes (i.e. undifferentiated hemocyte precursors) exist in circulation, they must be few. This problem must be properly discussed, and the "prohemocyte" terminology may be misleading.

RE) The prohemocyte (PH) cluster is characterized by the high expression of *Ance* or *Tep4* and depleted *Hml* or *Pxn* expressions, and it is the second most frequent cell type defined in lymph gland samples (**Reviewer's Fig. 5d**). Of the total number of PH cells, 69.33% originated from lymph glands, amounting to 16,554 out of 23,878 cells. The majority of PH cells in circulating hemocytes (72.24% of 6368) were identified in wasp-infected larvae (**Reviewer's Fig. 6**). For example, PH cells were identified in circulating hemocytes of wasp-infected larvae 96 h after egg laying (**Supplementary Fig. 1a**, right), and these cells may have originated from disintegrating lymph glands under the severe immune challenge.

Reviewer's Figure 6

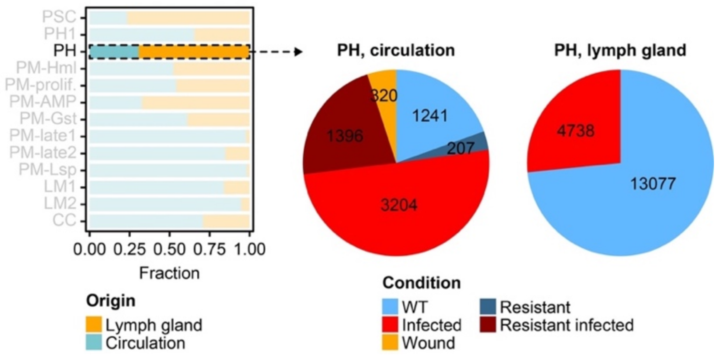

Reviewer's Figure 6. Proportions of sampling conditions in the PH cluster

The follow up on the *CG8501* marker is very interesting. Why is this important marker not displayed in Fig. 1d, and why does the text describe it as specific for PM (120) cells? According to the flyscrna database it is a good marker for PM cells in general.
RE) As per the reviewer's suggestion, *CG8501* expression has been included in the marker gene plot in the revised manuscript (**Reviewer's Fig. 5c**; **Figs. 1e and 2c** in the manuscript). It is worth noting that the top five most significant markers, as defined using adjusted *P*-values in the previous figure, were used in this analysis.

On line 275, it is stated that "knock-down of *CG8501* did not change the mRNA expression of NimC1 (Supplementary Fig. 6d)", but the figure shows what looks like a highly significantly INCREASED expression of NimC1. Any comment?
RE) As pointed out by the reviewer, RNAi targeting *CG8501* resulted in a moderate to significant increase in *NimC1* mRNA expression (**Supplementary Fig. 8d**). However, this effect was not evident in the plasmatocyte membrane, as determined by anti-NimC1 staining (**Fig. 4c**). Furthermore, Western blot analysis revealed that NimC1 protein levels in *CG8501* RNAi-expressing hemocytes increased, contrary to our findings by immunohistochemistry (**Supplementary Fig. 8e**). Thus, we hypothesized that the observed increase in *NimC1* mRNA and accumulated NimC1 protein in hemocytes may be due to a mislocalization of NimC1 protein induced by the absence of CG8501. We have revised the manuscript to better describe these results.

The PH 1 subcluster is a very interesting, case. Unlike the main PH cluster, the PH 1 cells may well correspond to a true class of prohemocytes; it is a small class, and it has a convincing overlap with vertebrate hematopoietic precursors. The presence of this subcluster among the circulating hemocytes suggests that some prohemocytes may after all be present in that population. However, cells similar to PH 1 were never detected in the other published single-cell studies, not even in the paper by Tattikota et al. 2020. How come?
RE) Regarding the comment about PH1, it is indeed a rare class of hemocytes found in the lymph gland and circulation. Due to their rarity, their detection is dependent on the degree of clustering analysis. In our previous analysis, we first performed a clustering analysis to identify broad cell types, then performed a subclustering analysis for each broad cell type to detect rare cells. However, it may be challenging to separate rare cells in general scRNA-seq analyses, because features or marker genes showing

high variance in those rare cells must be selected in the variable gene selection step. To address this issue, we used annotation by prediction—for example, through label transfer analysis—as a simple solution because prediction using these methods is independent of clustering and solely based on the similarities between transcriptomes. We were able to identify PH1 cells in other datasets by label transfer analysis, and these cells expressed PH1-specific marker genes, such as *Inx2* or *zld*, defined by our scRNA-seq analysis, indicating their presence in lymph gland and circulating hemocytes (**Reviewer's Fig. 7**). However, the number of cells in each dataset was very small, suggesting that PH 1 cells were missed during clustering analyses (**Reviewer's Fig. 2b**, Cattenoz et al., *n* = 2; Fu et al., *n* = 10; Leitão et al., *n* = 35; Girard et al., *n* = 1; Tattikota et al., *n* = 2).

Reviewer's Figure 7

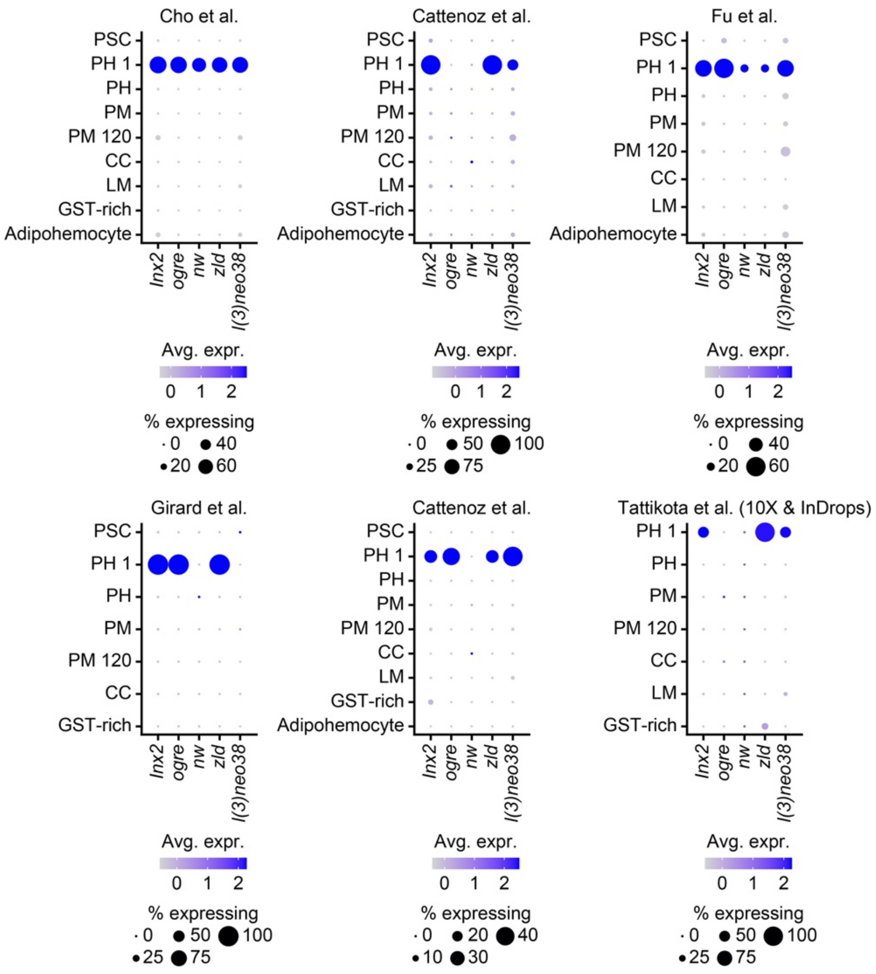

Reviewer's Figure 7. Dot plots of PH 1 marker gene expression in public scRNA-seq datasets

By the way, are the PH 1 cells included among the cells of the PH cluster, or should I understand these categories as mutually exclusive?

RE) PH1 was originally identified as a small group of cells in the PH cluster. We separated the PH1 cluster from the rest of the PH cells in our analysis, as it expressed a unique set of genes distinguishable from other PH cells (**Reviewer's Fig. 5c** and **7**).

Another discrepancy involves the hemocytes related to the cells of the posterior signaling center (PSC). Most of the previous studies found a well-defined class of such cells among the circulating cells, corresponding to a separate hemocyte type, dubbed "primocytes" by Fu et al. 2020. This class was also standing out in the data of Tattikota et al. 2020 (the "PM11" subcluster), but although the same data are included in the present manuscript, the "PSC" cluster is not represented among the circulating hemocytes. Why not?

RE) Initially, we removed the "PSC" cluster from circulating cells in our previous manuscript as we hypothesized that PSC cells from the lymph gland might have been mistakenly included as an artifact. However, recent papers have repeatedly identified PSC-like cells (or primocytes) in circulating hemocytes. Now, we have included predictions of this cell type in our study. By re-analyzing our Drop-seq data and three other scRNA-seq public datasets that sequenced circulating hemocytes (Cattenoz et al., Fu et al., and Leitão et al.), we identified small PSC-like clusters (*n* = 29, Cattenoz et al.; *n* = 9, Fu et al.; *n* = 14, Leitão et al.; *n* = 42, Drop-seq of Tattikota et al.) in circulating cells from all datasets. These clusters expressed well-known PSC markers, such as *CG15550*, *kn,* or *Antp*, suggesting that these cells are *bona fide* PSC-like hemocytes (**Reviewer's Fig. 5c** and **8**).

Reviewer's Figure 8

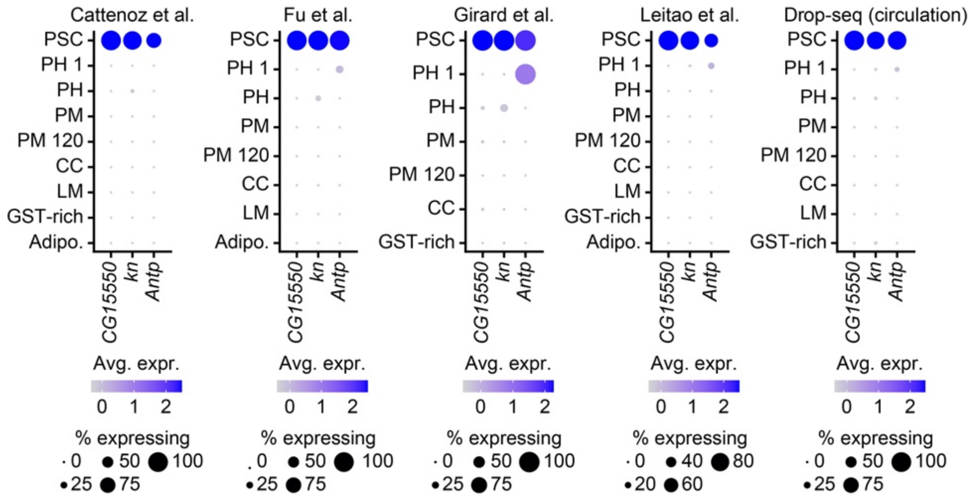

Reviewer's Figure 8. Dot plots of PSC marker gene expression in public scRNA-seq datasets

The "PM11" subcluster defined by Tattikota et al. expresses PSC-specific marker genes, such as *CG15550*, *ImpL2*, or *Antp*. However, only a small percentage of cells (24–27%) in the subcluster expressed these markers (Supplementary Table 2 in Tattikota et al., included in **Reviewer's Table 2**). Our analysis suggests that PSC-like cells in this subcluster were missed in the InDrops and 10X datasets, and only 42 cells were defined as PSC-like in the Drop-seq data of the circulating hemocytes in wasp-infected larvae (**Reviewer's Fig. 2**). Label transfer prediction from our data to Cattenoz et al., showed that the PL-ImpL2 subtype was well-correlated to the PSC cluster, and vice versa. However, this relationship was not found between PL-ImpL2 (Cattenoz et al.) and PM11 (Tattikota et al. without Drop-seq), indicating that PM11 is a mixture of PM and a small fraction of PSC-like cells.

gene	p_val	avg_logFC	pct.1	pct.2	p_val_adj
CG15550	1.87×10^{-281}	4.22	**0.26**	0.005	1.94×10^{-277}
CG6023	1.74×10^{-26}	2.75	0.24	0.054	1.81×10^{-22}
mthl7	0	2.61	0.17	0	0
tau	0	1.78	0.25	0.001	0
ImpL2	1.40×10^{-83}	1.75	**0.27**	0.023	1.46×10^{-79}
Antp	0	1.69	**0.24**	0.004	0
CG30054	1.92×10^{-123}	1.38	0.19	0.008	1.99×10^{-119}
kn	4.47×10^{-135}	1.38	**0.23**	0.01	4.64×10^{-131}
CG44325	6.40×10^{-32}	1.31	0.28	0.061	6.65×10^{-28}
CG9451	4.83×10^{-25}	1.31	0.10	0.011	5.02×10^{-21}

Reviewer's Table 2. Top 10 markers of PM11 reported by Tattikota et al.

The central part of this study involves the comparison between blood cell types (and states) in Drosophila and vertebrates. The most consistent relationship shown here is between Drosophila PH 1 cells and various vertebrate hematopoietic stem cells or precursors. This makes much sense, and I look much forward to future characterization (and confirmation) of the PH 1 class.

RE) PH1 was originally defined as a stem-like population in lymph glands. However, our study confirmed that this cell type was also found in circulating hemocytes regardless of sequencing platforms or conditions (**Reviewer's Fig. 7**). PH1 expressed a unique set of marker genes and showed similarities with hematopoietic stem cells in zebrafish or granulocyte progenitors in mice in our comparative analysis. This cell type could serve as a small reservoir to replenish hemocytes under stress conditions or for future use. We wish to conduct further research to better understand PH1 in the future.

Other correlations between the transcriptomes of Drosophila and vertebrate cell types are more uncertain. In general, they tend to link Drosophila hemocyte types to different vertebrate myeloid cells, but in some cases also to lymphoid cells. These correlations should be taken with several grains of salt. Lamellocytes are for instance strongly linked to Zebrafish and mouse neutrophils but to human monocytes Fig. 8). It should be noted that lamellocytes have only been found in a few Drosophila species, all closely related to D. melanogaster. In other Drosophila species they are replaced by other effector cell types, like the giant cells in D. ananassae. The transcriptomic profile of the latter cells is not very similar to that of D. melanogaster (Cinege et al. 2022). Similarly, the suggested relationship between crystal cells and Zebrafish NK/T cells or mouse "pDCs" (=plasmacytoid dendritic cells?) seems unlikely (Fig. 8). A relationship between plasmatocytes and mouse monocytes, or plasmatocytes (120 h) with macrophages or monocytes (Fig. 8) seems more likely, by the criterion of making sense. The value of this study is to point to similarities like these, but it should be pointed out that they do not necessarily imply homology (=common origin), rather than similar function. It could be speculated that ancestral blood cells had a phagocytic function, and that a phagocytic machinery has been retained in different more specialized blood cell types as well as in various "non-professional" phagocytes.

RE) We agree with the reviewer's concerns regarding the unexpected similarity between lamellocytes and neutrophils, which was relatively strong compared to others.

While we identified a few marker genes expressed in lamellocytes, we found that the expressions were less conserved in vertebrate myeloid cells (**Reviewer's Fig. 9**). Therefore, in the revised manuscript, we have removed our descriptions related to lamellocytes. Given that lamellocytes are a specialized cell type that functions to oppose parasitic infection in *Drosophila melanogaster*, the inferred relationship in this study may cause confusion. Although we could not provide convincing evidence for this similarity, we hope to characterize more about the relationship between these cell types in future studies.

Reviewer's Figure 9

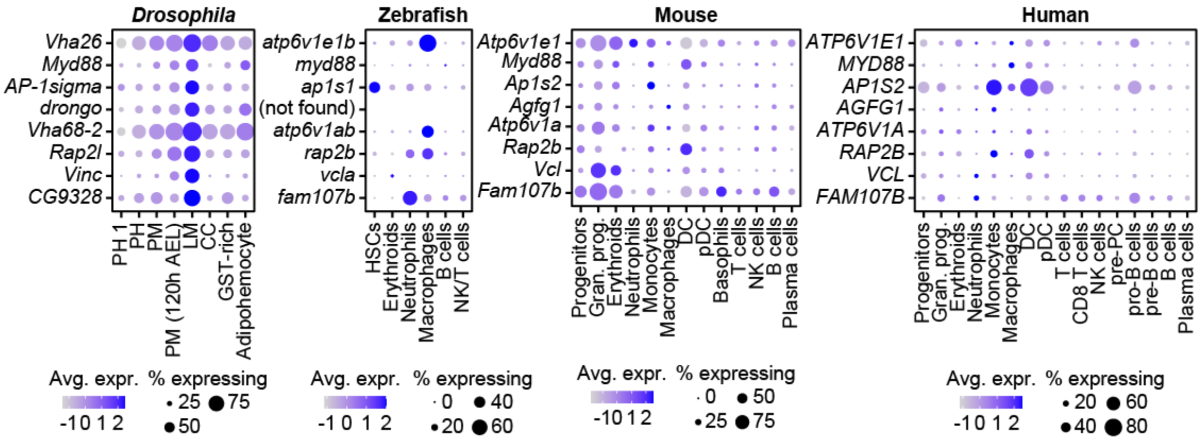

Reviewer's Figure 9. Dot plots of lamellocyte marker gene expression in four species

Regarding the comparisons between Drosophila and vertebrate blood cells I don't understand why the Drosophila transcriptomes were directly compared only with zebrafish. Mouse and human data were only secondarily compared with the zebrafish (Fig. 4 and Suppl. Figure 7). Direct comparisons between Drosophila and mouse or Drosophila and human were only shown in Fig. 8, although the latter was supposedly based on the comparisons in Fig. 4 and Suppl. Figure 7.

RE) To clarify the results, we now include all the comparative analyses in the new **Supplementary Fig. 9d** and **e** (shown below). Although we had compared *Drosophila* to mouse and human data, we did not include it in the previous manuscript.

Figure S9

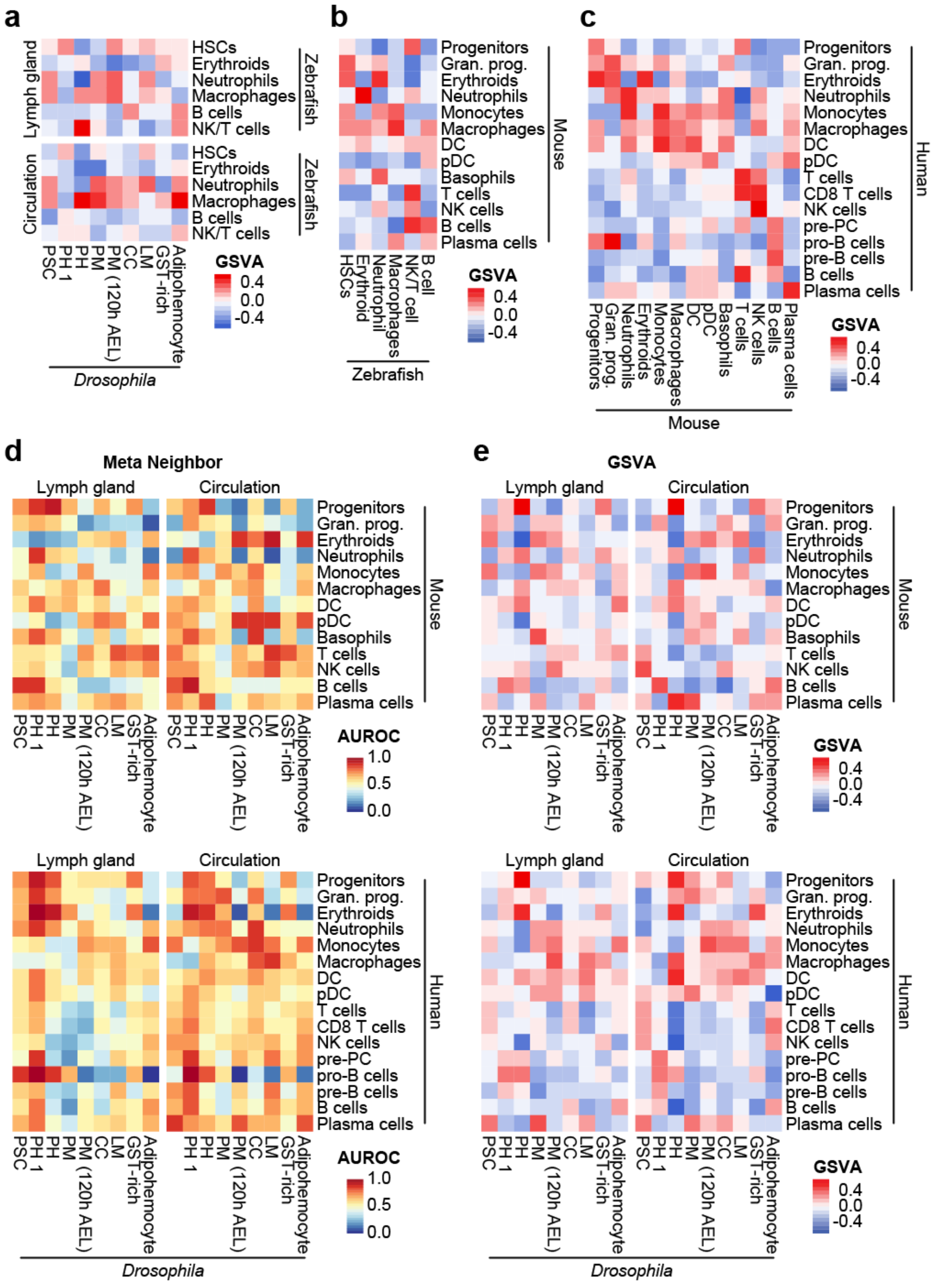

In conclusion, this is an important piece of work, trying for the first time to use transcriptomic data to identify relationships between blood cell types in insects and vertebrates. Novel findings include the possible existence of a prohemocyte class (PH 1 but, in my opinion, not PH in general) and the possible role of the CG8501 protein, but the uncertainties are not sufficiently emphasized, and the discrepancies between this study and those done elsewhere must be mentioned and discussed.

Minor points:

As far as possible, abbreviations should always be avoided. They tend to make reading unnecessarily difficult for anyone outside the particular narrow field. Newcomers quickly loose track, and the space you save is insignificant. Specifically, when cell types are discussed, their full names (plasmatocytes, lamellocytes etc.) should be fully spelled out. However, terms like PL, LM etc. are acceptable as designations of transcriptomic clusters (which are not necessarily synonymous with the established cell types). Abbreviations like LG for lymph gland are completely unnecessary in the main text.

RE) In the revised manuscript, we spelled out biological terms, such as lymph gland (LG) and plasmatocyte (PM).

The word infest is used for animals and pests that invade an area or space, like in a house infested with rats. For parasites, viruses and bacteria that affect an organism, the word infect is better. What is "steady state" (Fig. 1b). Does it mean uninfected?

RE) As per the reviewer's suggestion, we have changed "infested" to "infected" throughout the manuscript. Additionally, we have replaced "steady state" with "wild type" or "WT" to avoid any confusion.

The resolution is too low in some figures. For instance, in Fig. 1a it is not possible to see the dots corresponding to some of the cell types. Other figures have the same problem. In Fig. 2f and g, I am unable to read the text.

RE) We have increased the font size and panels to ensure better readability.

Reviewer #3:

In the current study by Yoon et al., entitled “Molecular Traces of Drosophila Hemocyte Evolution”, the authors have attempted a comprehensive cross species analysis between immune cells of Drosophila and vertebrate immune cells by employing the use of available single cell RNA seq data sets. As the authors compared the transcriptome of fly, fish, mouse and human immune cells, the data presented reveals common and distinguishing attributes of the respective Drosophila immune cells. Overall, through this approach the findings allude to:

1. homology of the fly immune cells to innate immune cells of vertebrates.
2. The data compares specific PH1 subset of Drosophila immune cells and reveals that this subset of prohemocyte was closest to hematopoietic progenitors and erythroid population.
3. the majority of Drosophila immune cells, which are plasmatocytes, are akin to macrophages and interestingly, the lamellocytes bear homology to neutrophils.
4. The authors also validate/annotate CG8501, which was found to be homologous to human CD59, to be important for phagocytic activity by regulating Hml and NimC1 in Drosophila.

Overall, the findings of the manuscript reveal a trend observed in immune cells of Drosophila. The large similarity of Drosophila immune cells with cells of zebrafish at a transcriptomic level is indeed intriguing. While I find the manuscript of substantial interest, but I am afraid the current draft and the manner in which it is drafted, do not deliver the information and the relevance of the analysis. My main concern is that the title of the manuscript, which is very broad and an ambitious one, but the contents of the manuscript in its current state fall short in delivering the same. The description of the data in the results section is very minimalistic when compared along side the figures, which are very elaborate. The figures are out of proportion with respect to the results section. The discussion as well, is very loose and does not really make a case for why this study is relevant for the field.

The data presented in the current state only proves the homology of fly immune cells to vertebrate myeloid lineage. While I agree this confirmation is good, but any point beyond this already established knowledge, any new additional understanding that

would prove the value of Drosophila immune cells as a powerful system and relevant towards understanding vertebrate myeloid physiology is not presented or discussed sufficiently. The draft falls short in presenting the data to highlight the value of their analysis. I feel that an analysis of such a kind should enable the field with a much deeper understanding of the Drosophila immune system and empower it further to be used as a tool to address questions relevant to myeloid physiology. The finding and representation of a handful of genes with only, CG8501 and its homology with vertebrate immune cells does not sufficiently prove "Molecular Traces of Drosophila Hemocyte Evolution". I am therefore afraid the draft in its current state does not deliver this message.

I strongly urge the authors to re-write the manuscript to elaborate and provide more detail on the data and better discussion of genes or classes that would provide newer substance and information, which is beyond proving our current knowledge. The values of cross comparing 4 model systems with details on the obtained information with a well-bodied discussion that would further empower Drosophila as a key model system to uncover myeloid physiology and function is what I strongly recommend.

RE) We thank the reviewer for their insightful comments. As per the reviewer's suggestions, we have made extensive modifications to the results and discussion sections and have updated the title of the manuscript. Please find detailed changes below.

There are also a few minor comments for the author to be addressed:
1) Fig S1a: In the methods it is mentioned that for the scRNA seq 100 larvae were taken, if that is correct then circulating hemocytes in steady state in all the developmental conditions is underrepresented, which may incorporate biases in data interpretation.
RE) It has been observed that a *Drosophila* larva contains around 5,000–10,000 hemocytes. For the one-time experiment, approximately $5*10^5$–$1*10^6$ hemocytes from 100 larvae were used. We should admit that the cell counts collected for this experiment were not as great as those found in other circulating hemocyte scRNA seq datasets, including that of Tattikota et al. However, upon comparing the circulating hemocyte scRNA-seq data to previous datasets, such as Tattikota et al., it was found

that the sequencing data was enough to cover all the hemocyte subpopulations that were described in previous analyses. Therefore, we concluded that this does not impart any biases to the data interpretation. We now have included additional explanations in the methods.

2) In the manuscript author claims that CG8501RNAi do not impact the total hemocytes but significantly impact the major hemocyte population. This is not supported by any compensation of other hemocyte population.

RE) In **Fig. 4d** and **e**, we demonstrated that the expression of *CG8501* RNAi significantly reduced the number of Hml^+ plasmatocytes compared to wild-type controls. However, this reduction in Hml^+ plasmatocytes did not affect the total number of hemocytes indicated by DAPI or $PPO1^+$ crystal cells (**Supplementary Figure 8c**). This suggests that the loss of *CG8501* specifically alters the number of plasmatocytes expressing Hml (possibly PM-Hml). To probe this phenotype in detail, we applied an additional plasmatocyte marker, Pxn, and found that the number of Pxn^+ plasmatocytes remains unchanged in the *CG8501* RNAi expressing animals (**Supplementary Figure 8b** and **c**). The new data is included in the revised manuscript.

3) FigS6b: the graph seems to be out of place as in text author is addressing Plasmatocytes while quoting this graph, which to my understanding is representing crystal cell population.

RE) We apologize for our mistake. As pointed out by the reviewer, the graph, now in **Supplementary Figure 8c** indicates the number of $PPO1^+$ crystal cells. We have updated the description and figure citation accordingly in the revised manuscript.

4) FigS6c: In 2nd image of the figS6C one of the larvae do not show any reduction of Hml–UASGFP positive cells as claimed in the text, author need to change the image.

RE) We initially hypothesized that variations in Hml^+ hemocyte expression could be linked to RNAi efficiency. However, as pointed out by the reviewer, this image in question may not accurately support our claim. Therefore, we have made the necessary change in what is now **Figure 4f**.

5) Fig 3: Quantification for the NimC1 positive cells is missing alongside Hml positive cells, as it is one of the important finding highlighted by author, under CG8051RNAi condition.

RE) As per the reviewer's suggestion, we have now included quantification for the NimC1^{+} plasmatocytes in **Figure 4e.**

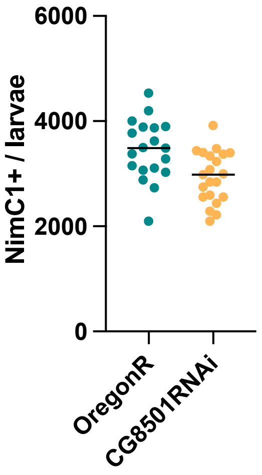

***Figure 4e**, right*

6) Fig 3c & d: Author highlights that CG8051RNAi reduces the NimC1 protein levels (through anti-NimC1 antibody) but figS6d shows high mRNA levels. With the understanding that mRNA levels need not always be correct proxy for protein levels. This point is raised/important because author has used the same data set to support the low Hml protein levels as mRNA levels of Hml are also low. But for the NimC1 the results are contrary.

RE) This is an important point raised by the reviewer. Our hypothesis is that the loss of CG8501 may lead to the improper trafficking or targeting of protein NimC1 in the membrane, subsequently leading to transcriptional feedback that increases the expression of *NimC1*. In our revised manuscript, we performed a Western blot analysis to assess NimC1 protein levels in hemocytes and revealed a consistent increase in NimC1 protein levels in larvae expressing *CG8501* RNAi (**Supplementary Fig. 8e**). Thus, we hypothesized that losing CG8501 leads to both the mislocalization of NimC1 proteins and an upregulation of its transcription. We now have included this hypothesis and revised the explanation accordingly.

7) even though we can clearly see that there is a significant increase in NimC1 mRNA levels in FigS6d, author is claiming no change and on this basis they are claiming CG8051 is important for stabilizing NimC1, therefore it needs more explanation.

RE) As per the reviewer's suggestion, we have modified the manuscript as explained in previous responses.

8) Hml is a common marker for all three blood cell type, author do address the impact on Plasmatocytes population with the help of NimC1 but what are the consequences of CG8051RNAi on crystal cell and lamellocytes is worth understanding.

RE) *Hml* is a marker for plasmatocytes despite its residual expressions in premature crystal cells or lamellocytes. We have shown that *CG8501* RNAi does not alter the differentiation of crystal cells (**Supplementary Figure 8c**). Furthermore, we could not observe abnormal lamellocyte differentiation caused by *CG8501* RNAi under unchallenged conditions, as shown in **Figure 4c**, **d** and **Supplementary Figure 8b**.

9) In material and methods infection strategy is missing.

RE) Wasp infection was performed in previous single-cell analysis studies but was not conducted in the current study.

10) In Drosophila hemocytes crystal cells are often compared functionally with platelets but this cross species analysis did not address this point, any comments on this aspect?

RE) As the reviewer mentioned, crystal cells are considered comparable to platelets in vertebrates due to their melanization capacity. However, we learned from our cross-species analysis that functional similarities do not always mean transcriptional homologies. We observed that crystal cells are rather similar to NK/T cells in zebrafish and hope that this observation can be substantiated by future studies.

Reference

1. Cattenoz PB, Sakr R, Pavlidaki A, Delaporte C, Riba A, Molina N, et al. Temporal specificity and heterogeneity of Drosophila immune cells. EMBO J. 2020;39(12):e104486. Epub 2020/03/13. doi: 10.15252/embj.2020104486. PubMed PMID: 32162708; PubMed Central PMCID: PMCPMC7298292.
2. Cho B, Yoon SH, Lee D, Koranteng F, Tattikota SG, Cha N, et al. Single-cell transcriptome maps of myeloid blood cell lineages in Drosophila. Nat Commun. 2020;11(1):4483. Epub 2020/09/10. doi: 10.1038/s41467-020-18135-y. PubMed PMID: 32900993; PubMed Central PMCID: PMCPMC7479620.
3. Fu Y, Huang X, Zhang P, van de Leemput J, Han Z. Single-cell RNA sequencing identifies novel cell types in Drosophila blood. J Genet Genomics. 2020;47(4):175–86. Epub 2020/06/04. doi: 10.1016/j.jgg.2020.02.004. PubMed PMID: 32487456; PubMed Central PMCID: PMCPMC7321924.
4. Girard JR, Goins LM, Vuu DM, Sharpley MS, Spratford CM, Mantri SR, et al. Paths and pathways that generate cell-type heterogeneity and developmental progression in hematopoiesis. Elife. 2021;10:e67516.
5. Leitao AB, Arunkumar R, Day JP, Geldman EM, Morin-Poulard I, Crozatier M, et al. Constitutive activation of cellular immunity underlies the evolution of resistance to infection in Drosophila. Elife. 2020;9. Epub 2020/12/29. doi: 10.7554/eLife.59095. PubMed PMID: 33357377; PubMed Central PMCID: PMCPMC7785293.
6. Tattikota SG, Cho B, Liu Y, Hu Y, Barrera V, Steinbaugh MJ, et al. A single-cell survey of Drosophila blood. Elife. 2020;9. Epub 2020/05/13. doi: 10.7554/eLife.54818. PubMed PMID: 32396065; PubMed Central PMCID: PMCPMC7237219.
